# A Somatic BRCA2-Mutated Pancreatic Adenocarcinoma With Sustained Exceptional Response to Modified FOLFIRINOX

**DOI:** 10.1093/oncolo/oyad315

**Published:** 2024-02-23

**Authors:** Jacob K Jamison, Michael S May, Alexander G Raufi, Lyndon Luk, Winston Wong, Prabhjot S Mundi, Gulam A Manji

**Affiliations:** Weill Cornell Medical College, New York, NY, USA; Department of Medicine, Vagelos College of Physicians and Surgeons, Columbia University, New York, NY, USA; Division of Hematology/Oncology, Department of Medicine, Lifespan Health System and Brown University, Providence, RI, USA; Herbert Irving Comprehensive Cancer Center, Vagelos College of Physicians and Surgeons, Columbia University, New York, NY, USA; Memorial Sloan Kettering Cancer Center, New York, NY, USA; Herbert Irving Comprehensive Cancer Center, Vagelos College of Physicians and Surgeons, Columbia University, New York, NY, USA; Department of Medicine, Vagelos College of Physicians and Surgeons, Columbia University, New York, NY, USA; Herbert Irving Comprehensive Cancer Center, Vagelos College of Physicians and Surgeons, Columbia University, New York, NY, USA

**Keywords:** pancreatic cancer, precision medicine, homologous recombination deficiency

## Abstract

Homologous recombination repair (HRR) pathway deficiency opens multiple therapeutic avenues within pancreatic cancer. Patients with HRR deficiency–associated gene mutations such as *BRCA1*, *BRCA2*, and *PALB2* are more susceptible to platinum-based chemotherapies and in those with somatic *BRCA* mutations, PARP inhibitor therapy prolongs progression-free survival. The case discussed herein illustrates the therapeutic opportunities offered through the identification of HRR deficiency in pancreatic cancer, as well as the challenges associated with treatment and prevention of central nervous system metastases in long-term survivors of pancreatic cancer.

## Teaser

This case report illustrates the therapeutic opportunities offered through identification of homologous recombination repair deficiency in pancreatic cancer, as well as the challenges associated with treatment and prevention of central nervous system metastases in long-term survivors of pancreatic cancer.

**Key Points**

- Metastatic pancreas adenocarcinoma is a uniformly fatal disease where palliative chemotherapy affords a limited survival benefit.
- However, in rare cases, a minority of patients experience exceptional responses.
- Herein we describe one such case of a patient who experienced prolonged benefit from modified FOLFIRINOX therapy and mutational analysis of the tumor identified a mutation in the homologous recombination pathway demonstrating the importance of conducting next-generation sequencing which may inform treatment choices in this disease.

## Introduction

Pancreatic ductal adenocarcinoma (PDAC) is an aggressive disease with a dismal 5-year overall survival rate of 12%.^1^ By 2030, PDAC is projected to overtake colon cancer to become the second-leading cause of cancer-related death in the US.^2^ First-line treatment for metastatic PDAC includes combination chemotherapy with either FOLFIRINOX (fluorouracil (5-FU), leucovorin, irinotecan, and oxaliplatin) or gemcitabine with nab-paclitaxel. However, the median overall survival achieved with these regimens remains under a year (11.1 vs 6.8 and 8.5 vs 6.7 months compared to gemcitabine alone, respectively), highlighting the need for more effective treatments.^3,4^

Despite frequent alterations in genes such as *KRAS*, *TP53*, *CDKN2A*, and *SMAD4,* the development of effective targeted therapies for PDAC is a persistent challenge.^5^ A promising step was seen with the *KRAS* G12C inhibitor sotorasib, showing a 21.1% objective response rate and 84.2% disease control rate in a phase I/II trial among patients with metastatic PDAC who had received at least one line of therapy, suggesting that KRAS in PDAC is not undruggable. However, the median progression-free survival was 4.0 months, indicating that resistant pathways dominate the disease quickly. Moreover, *KRAS* G12C mutations are found in only 1%-2% of pancreatic cancers.^6^ Germline mutations in homologous recombination repair (HRR) pathway genes such as *BRCA1*, *BRCA2*, and *PALB2* (approximately 5%-9% of PDAC) are more prevalent and have shown susceptibility to platinum compounds (Supplementary Table S1 shows additional references). Platinum-based chemotherapy’s efficacy in this setting has been attributed to the inability of homologous recombination deficient (HRD) tumor cells to repair DNA in the presence of platinum-induced DNA damage.^7^ The discovery that PARP inhibitors could also induce sustained responses in HRD PDAC led to the approval of the first oral agent for PDAC in decades.^8,9^ Somatic *BRCA* (s*BRCA*) mutations are also speculated to give rise to an HRD phenotype, and clinical trials investigating their sensitivity to platinum agents and PARP inhibitors are underway (Supplementary Table S2 shows the active clinical trials utilizing PARP inhibitor therapy in non-germline BRCA1/2 HRD PDAC).^10^

Here we report a patient with s*BRCA2*-mutated PDAC with metastases to the liver and brain who achieved a 60-month survival with modified FOLFIRINOX (mFOLFIRINOX) and brain lesion resection with Gamma Knife radiosurgery.

## Patient Story

**Figure 1.**
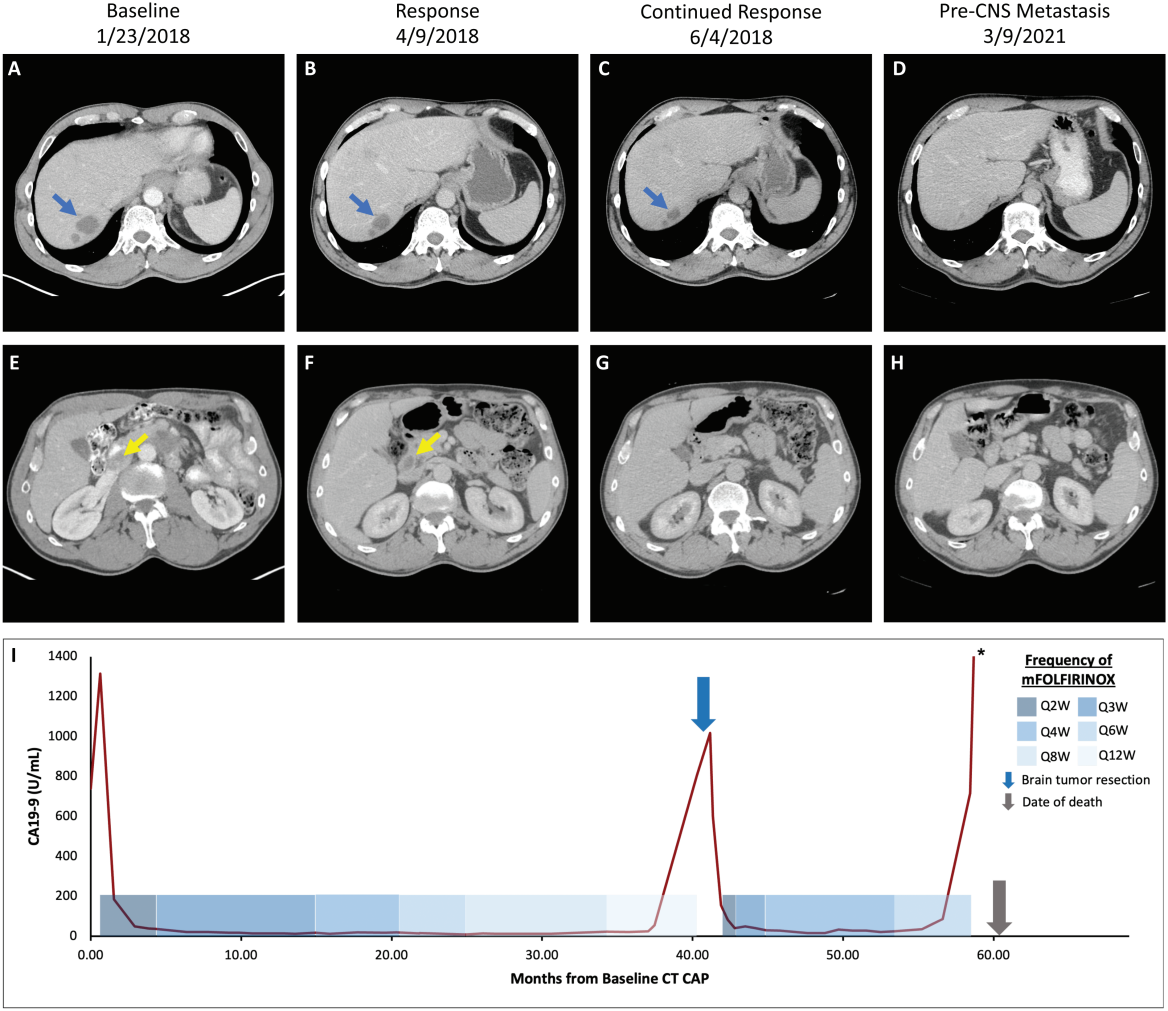
Radiological and biochemical response. Serial contrast-enhanced abdominal CT scans showing the patient’s baseline disease prior to initiation of mFOLFIRINOX (**A**, **E**), response to mFOLFIRINOX (**B**, **F**), continued response to mFOLFIRINOX (**C**, **G**), and sustained response 3 months prior to identification and resection of brain metastasis (**D**, **H**). Graphical depiction of CA19-9 levels over time (**I**) with response to mFOLFIRINOX. Yellow arrow: Primary pancreatic adenocarcinoma. Blue arrow: Metastatic pancreatic adenocarcinoma to hepatic segment VII. Abbreviations: CNS, central nervous system; mFOLFIRINOX, modified fluorouracil, leucovorin, irinotecan, oxaliplatin; CT CAP, computed tomography scan of chest, abdomen, and pelvis; Q, every; W, weeks. *CA19-9 value of 4762 U/mL.

A 57-year-old man presented with newly diagnosed PDAC with metastasis to the liver. Computed tomography (CT) of the abdomen and pelvis revealed a 3.8 × 2.7-cm uncinate mass in the pancreas which abutted the superior mesenteric artery and aortic wall (Fig. 1E). In addition, multiple liver lesions, the largest measuring 3.1 cm × 2.6 cm in segment 7 (Fig. 1A), were also noted. Endoscopic ultrasound-guided fine needle aspiration of both the pancreas and the liver mass confirmed the diagnosis. Immunohistochemistry (IHC) staining and somatic mutation analysis were not performed due to insufficient tissue. Germline testing (Ambry Genetics) identified no pathogenic mutations or variants of unknown significance. The patient elected to initiate therapy with mFOLFIRINOX and experienced significant improvement in abdominal pain after first cycle of therapy.

CT scan after 4 cycles of mFOLFIRINOX revealed a partial response within the pancreatic mass (1.8 × 1.3 cm) and segment 7 liver lesion (2.6 × 1.9 cm) (Fig. 1B, 1F). Repeat CT scan performed after 8 cycles revealed no identifiable pancreatic mass and continued shrinkage of the hepatic lesions, with the segment 7 lesion decreasing in size to 2.0 × 1.3 cm (Fig. 1C, 1G). At this time, the patient’s serum carbohydrate antigen (CA) 19-9 had decreased from 1314 U/mL at baseline to 36 U/mL (Fig. 1I). Imaging after 12 cycles revealed further shrinkage of the segment 7 liver lesion (1.7 × 1.6 cm) and resolution of all other hepatic metastases. In the setting of continued radiographic and biochemical response, increased fatigue, and patient preference, the intervals between his treatments over the next 2 years were progressively extended. By cycle 37, he was being treated every 12 weeks (Fig. 1I).

**Figure 2.**
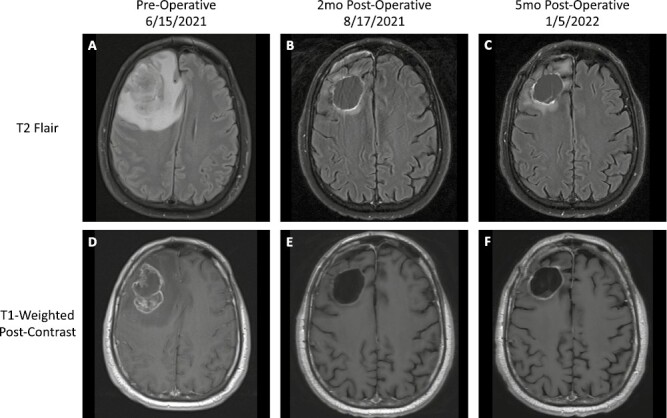
Disease progression within the brain. T2 FLAIR and T1-weighted postcontrast MRI scans showing the patient’s brain mass at presentation (**A**, **D**), 2 months following resection (**B**, **E**), and 5 months following resection (**C**, **F**).

After 40 months (39 cycles) of mFOLFIRINOX, the patient presented to a local emergency department with acute onset of left facial droop, headache, and altered mental status. Magnetic resonance imaging (MRI) of the brain and spine identified a heterogeneously enhancing 5.2 × 3.4 cm right frontal lobe mass (Fig. 2A, 2D), as well as T8 and L2 vertebral lesions suspicious for metastasis. Emergent right frontal lobe resection followed by Gamma Knife radiosurgery (GKRS) was performed on the frontal lobe mass which on pathological review was consistent with metastasis from the pancreas (Table 1). Results of the next-generation sequencing (NGS; FoundationOne CDx, 2021) performed on the central nervous system (CNS) tumor demonstrated a pathologic s*BRCA2*^S2219*^ mutation (Table 1).

**Table 1.** Complete pathological staining and next-generation sequencing results for the patient’s resected brain tissue.

IHC stain	Expression on resected brain tissue
CK-7 [^11-13^]	Negative
CK-20 [^11-13^]	Negative
GATA3	Negative
MUC2 [^14,15^]	Negative
NKX3-1	Negative
NTRK	Negative
PAX8	Negative
TTF-1	Negative
MSI	Negative
MMR	Preserved
Cytokeratin, pan	Positive
CDX-2 [^16^]	Positive
MUC1b [^14,15^]	Positive (diffuse, with intense staining)
MUC5 [^14,15^]	Positive (diffuse, with intense staining)
MUC6 [^15^]	Positive (patchy subset, with moderate to focally intense staining)
SATB2	Positive (diffuse, with weak to moderate nuclear staining)
HER2/neu amplification	Positive
PD-L1	Positive (CPS > 1)

Foundation one—gene identified	Mutation
BRCA2	S2219*
ERBB2	Amplification
KRAS	G12R
NF2	E204*
TP53	F113C

Abbreviations: MSI, microsatellite instability; MMR, mismatch repair; CPS, combined positive score.

Patient’s CA19-9 was 802 U/mL prior to the frontal lobe resection, 1018 U/mL 13 days after resection, and 598 U/mL prior to resumption of mFOLFIRINOX (Fig. 1I). Given the previous treatment response despite prolonged intervals between cycles of mFOLFIRINOX, he was restarted on biweekly chemotherapy. This approach was weighed against considering olaparib, which may have offered higher levels of CNS penetration at the potential risk of not providing the profound systemic efficacy seen with mFOLFIRINOX.^17^ Additionally, data on the sensitivity of s*BRCA*-mutated PDAC to targeted therapy were limited, and clinical trials largely excluded patients with brain metastasis.

MRI of the brain demonstrated a resolving resection cavity and no evidence of disease recurrence (Fig. 2B, 2E). While receiving mFOLFIRINOX, an olfactory groove lesion was identified 5 months postresection and was treated with GKRS (Fig. 2C, 2F). CA19-9 continued to decline and treatment was attenuated (dose and schedule) per patient preference until 18 months postresection (Fig. 1I), at which time MRI revealed new leptomeningeal disease with rising CA19-9. Patient received proton craniospinal irradiation followed by 1 week of olaparib, during which time CA19-9 further increased to 4762 U/mL (Fig. 1I). He subsequently decided to withhold all cancer-directed therapy and pursue best supportive care due to overwhelming fatigue and deterioration in performance status.

## Molecular Tumor Board

Sustained complete or near-complete response to systemic chemotherapy is rare in metastatic PDAC, highlighting the need for improved patient selection using predictive biomarkers (Supplementary Table S1). HRR deficiency is a prominent example, predicting response to both platinum-based chemotherapy and PARP inhibitors.^8,18^ While observed most frequently in breast and ovarian cancers, the prevalence of HRR deficiency has been estimated to range from 5% to 9% to as high as 19% in PDAC (Supplementary Table S1). Germline (g)*BRCA1/2* mutations have been reported in 1%-8.1% of patients with PDAC, whereas the prevalence of s*BRCA1/2* mutations is estimated to affect 4.7% (1.4% for s*BRCA1*, 3.3% for s*BRCA2*; Supplementary Table S1).

The phase III POLO trial demonstrated that maintenance therapy with the PARP inhibitor olaparib significantly prolonged progression-free survival in patients with g*BRCA1/2*-mutated PDAC when compared to placebo (7.4 vs 3.8 months). The study noted 2 complete radiological responses in the treatment group.^9^ Recent data suggest that somatic mutations confer a similar sensitivity to PARP inhibition. A meta-analysis found similar progression-free survival and response rates with PARP inhibition for solid tumors harboring germline versus s*BRCA* mutations (43.9% vs 55.8%, respectively, across all tumor types and with 43/377 patients representing PDAC cases).^19^ Complete responses, although rare, have been reported in both g*BRCA1/2* and s*BRCA1/2* mutated PDAC treated with PARP inhibition (Supplementary Table S1). Research is ongoing to determine whether mutations in other non-g*BRCA1/2* HRR genes can predict response to PARP inhibitors (Supplementary Table S2).

Mutations in the HRR pathway may also predict response to treatment with platinum-based chemotherapy (Supplementary Table S1). A phase II trial of gemcitabine and cisplatin in PDAC patients harboring g*BRCA1/2* or g*PALB2* mutations reported a response rate of 65.2%.^18^ A recent cohort study of *BRCA/PALB2*-mutated PDAC (determined via NGS from tumor tissue) found similar response rates with the administration of FOLFIRINOX (70.0% in *BRCA/PALB2*-mutated compared to 22.1% in wild-type PDAC).^20^ Another recent study found comparable response rates to first-line platinum therapy between *sBRCA1/2*-mutated (6/7 patients, 86%) and *gBRCA1/2*-mutated (46/58, 79%) PDAC.^21^ Despite impressive overall response rates, complete responses to platinum-based therapy in HRD PDAC are extraordinarily rare (Supplementary Table S1).

**Figure 3.**
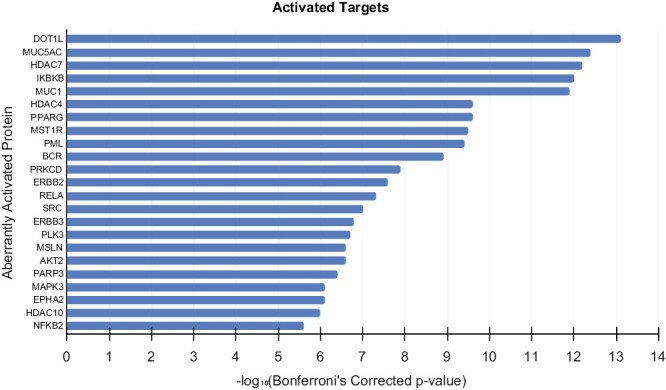
Transcriptomic analysis of resected brain metastasis. Transcriptome analysis of resected brain tissue depicting aberrantly activated genes based on Bonferroni’s corrected *P*-value calculated with Darwin OncoTarget.

To identify other potential targets for the treatment of this patient, we performed whole transcriptome sequencing on the patient’s resected metastatic brain tumor tissue using Darwin OncoTarget. Sequencing was not performed on metastatic liver tissue due to insufficient tumor material. OncoTarget uses the Virtual Inference of Protein activity by Enriched Regulon analysis (VIPER) algorithm to infer protein activity from a tumor’s transcriptomic signature.^22^ This identified aberrant activation in multiple drivers, highlighting the complexity of identifying key pathways to therapeutically target, particularly in the context of a patient with a previously established sensitivity to chemotherapy (Fig. 3).

Chemotherapy, however, rarely controls cancer that has spread to sanctuary sites such as the CNS.^23^ Notably, brain metastases occur in <1% of patients with PDAC and predict worse survival.^24,25^ Additionally, *BRCA* mutations are associated with higher rates of brain metastasis in breast, ovarian, and pancreatic cancers (Supplementary Table S1). In such circumstances of oligometastatic disease, if deemed safe, surgery or radiotherapy is preferred.^26^ Small-molecule drugs are thought to achieve improved blood-brain barrier (BBB) penetration, and studies of new approaches to expand delivery to brain metastases are ongoing.^27,28^ Our patient was eventually briefly treated with olaparib, a small-molecule agent with known PARP3 inhibition and demonstrated efficacy in s*BRCA2-*mutated PDAC and other solid tumors without CNS disease.^29,30^ Olaparib has resulted in tumor responses within CNS metastasis from breast and ovarian cancers, and was detected within glioblastoma when measured by liquid chromatography mass spectrometry (Supplementary Table S1).^17^ Improved outcomes with PARP inhibitors compared to historical controls treated with standard chemotherapy have been reported in patients with breast and ovarian cancer whose cancers have metastasized to the CNS (Supplementary Table S1). It is unclear whether the introduction of olaparib earlier would have benefited the patient’s outcome. Paucity of evidence regarding the effectiveness of PARP inhibitors during the first few years of treatment with mFOLFIRINOX and the exceptional tumor control with schedule-attenuated chemotherapy were the reasons that olaparib was not introduced sooner. Moreover, little is known about the distinct ways that different cancers compromise the BBB and how this may affect response to PARP inhibition.^31^

This case underscores the substantial treatment benefit that PDAC patients with HRR deficiency can experience, as well as the limitations of chemotherapy in long-term survivors. The patient’s s*BRCA* nonsense mutation likely conferred a dramatic, sustained response to mFOLFIRINOX (particularly oxaliplatin). Most therapies that prolong survival, however, cannot cross the BBB and may lead to sanctuary sites such as the CNS becoming favored sites of metastasis. Therefore, significant efforts should be made in considering agents with CNS penetrance, as they have the potential to prolong survival. Given the multitude of identified mutations yet paucity of effective therapies, the role of somatic tumor testing and transcriptome analysis in expanding the treatment armamentarium in PDAC warrants continued exploration. Although it is difficult to assess when a sanctuary site may be seeded with metastatic disease, molecular methods could help guide treatment expansion to include BBB-penetrating drugs, especially in those who are at greater risk of CNS disease.

## Supplementary Material

Supplementary material is available at *The Oncologist* online.

oyad315_suppl_Supplementary_Tables_S1

oyad315_suppl_Supplementary_Tables_S2

## Data Availability

The data underlying this article cannot be shared publicly due to patient confidentiality. The data will be shared on reasonable request to the corresponding author.
