# Supplementary material for: A Somatic BRCA2-Mutated Pancreatic Adenocarcinoma With Sustained Exceptional Response to Modified FOLFIRINOX
Source: Oncologist. 2024 Feb 23;29(4):350–5. doi: 10.1093/oncolo/oyad315 (PMC10994267; doi:10.1093/oncolo/oyad315)
Supplement: oyad315_suppl_Supplementary_Tables_S1 [file oyad315_suppl_supplementary_tables_s1.pdf]

Prevalence of HRD in PDAC	Nguyen, L., W M Martens, J., Van Hoeck, A., & Cuppen, E. (2020). Pan-cancer landscape of homologous recombination deficiency. Nature communications, 11(1), 5584. https://doi.org/10.1038/s41467-020-19406-4<br>Park, W., Chen, J., Chou, J. F., Varghese, A. M., Yu, K. H., Wong, W., Capanu, M., Balachandran, V., McIntyre, C. A., El Dika, I., Khalil, D. N., Harding, J. J., Ghalehsari, N., McKinnell, Z., Chalasani, S. B., Makarov, V., Selenica, P., Pei, X., Lecomte, N., Kelsen, D. P., … O'Reilly, E. M. (2020). Genomic Methods Identify Homologous Recombination Deficiency in Pancreas Adenocarcinoma and Optimize Treatment Selection. Clinical cancer research : an official journal of the American Association for Cancer Research, 26(13), 3239–3247. https://doi.org/10.1158/1078-0432.CCR-20-0418<br>Singhi, A. D., George, B., Greenbowe, J. R., Chung, J., Suh, J., Maitra, A., Klempner, S. J., Hendifar, A., Milind, J. M., Golan, T., Brand, R. E., Zureikat, A. H., Roy, S., Schrock, A. B., Miller, V. A., Ross, J. S., Ali, S. M., & Bahary, N. (2019). Real-Time Targeted Genome Profile Analysis of Pancreatic Ductal Adenocarcinomas Identifies Genetic Alterations That Might Be Targeted With Existing Drugs or Used as Biomarkers. Gastroenterology, 156(8), 2242–2253.e4. https://doi.org/10.1053/j.gastro.2019.02.037<br>Casolino, R., Paiella, S., Azzolina, D., Beer, P. A., Corbo, V., Lorenzoni, G., Gregori, D., Golan, T., Braconi, C., Froeling, F., Milella, M., Scarpa, A., Pea, A., Malleo, G., Salvia, R., Bassi, C., Chang, D. K., & Biankin, A. V. (2021). Homologous Recombination Deficiency in Pancreatic Cancer: A Systematic Review and Prevalence Meta-Analysis. Journal of clinical oncology : official journal of the American Society of Clinical Oncology, 39(23), 2617–2631. https://doi.org/10.1200/JCO.20.03238<br>Heeke, A. L., Pishvaian, M. J., Lynce, F., Xiu, J., Brody, J. R., Chen, W. J., Baker, T. M., Marshall, J. L., & Isaacs, C. (2018). Prevalence of Homologous Recombination-Related Gene Mutations Across Multiple Cancer Types. JCO precision oncology, 2018, PO.17.00286. https://doi.org/10.1200/PO.17.00286
Sustained complete or near-complete response to systemic chemotherapy in metastatic PDAC	Christos Nikolaou, Alexios Matikas, Maria Papavasilopoulou, Dimitris Mavroudis, Lampros Vamvakas, "Prolonged Complete Response in a Patient with Metastatic Pancreatic Adenocarcinoma after FOLFIRINOX Chemotherapy and Maintenance with FOLFIRI", Case Reports in Oncological Medicine, vol. 2015, Article ID 659624, 4 pages, 2015. https://doi.org/10.1155/2015/659624<br>Shakir, A. R. (2014). A Near-Complete Response to Treatment with Gemcitabine plus nab-Paclitaxel in a Patient with Metastatic Pancreatic Cancer and Poor Performance Status: A Case Report. Case reports in oncology. 7. 711-717. https://doi.org/10.1159/000368346<br>Shelemey, P. T., Amaro, C. P., Ng, D., Falck, V., & Tam, V. C. (2021). Metastatic pancreatic cancer with complete response to FOLFIRINOX treatment. BMJ case reports, 14(5), e238395. https://doi.org/10.1136/bcr-2020-238395
Prevalence of *BRCA* mutations in PDAC	Casolino, R., Paiella, S., Azzolina, D., Beer, P. A., Corbo, V., Lorenzoni, G., Gregori, D., Golan, T., Braconi, C., Froeling, F., Milella, M., Scarpa, A., Pea, A., Malleo, G., Salvia, R., Bassi, C., Chang, D. K., & Biankin, A. V. (2021). Homologous Recombination Deficiency in Pancreatic Cancer: A Systematic Review and Prevalence Meta-Analysis. Journal of clinical oncology : official journal of the American Society of Clinical Oncology, 39(23), 2617–2631. https://doi.org/10.1200/JCO.20.03238<br>Heeke, A. L., Pishvaian, M. J., Lynce, F., Xiu, J., Brody, J. R., Chen, W. J., Baker, T. M., Marshall, J. L., & Isaacs, C. (2018). Prevalence of Homologous Recombination-Related Gene Mutations Across Multiple Cancer Types. JCO precision oncology, 2018, PO.17.00286. https://doi.org/10.1200/PO.17.00286<br>Holter, S., Borgida, A., Dodd, A., Grant, R., Semotiuk, K., Hedley, D., Dhani, N., Narod, S., Akbari, M., Moore, M., & Gallinger, S. (2015). Germline BRCA Mutations in a Large Clinic-Based Cohort of Patients With Pancreatic Adenocarcinoma. Journal of clinical oncology : official journal of the American Society of Clinical Oncology, 33(28), 3124–3129. https://doi.org/10.1200/JCO.2014.59.7401<br>Rosen, M. N., Goodwin, R. A., & Vickers, M. M. (2021). BRCA mutated pancreatic cancer: A change is coming. World journal of gastroenterology, 27(17), 1943–1958. https://doi.org/10.3748/wjg.v27.i17.1943<br>Lowery, M. A., Wong, W., Jordan, E. J., Lee, J. W., Kemel, Y., Vijai, J., Mandelker, D., Zehir, A., Capanu, M., Salo-Mullen, E., Arnold, A. G., Yu, K. H., Varghese, A. M., Kelsen, D. P., Brenner, R., Kaufmann, E., Ravichandran, V., Mukherjee, S., Berger, M. F., Hyman, D. M., … O'Reilly, E. M. (2018). Prospective Evaluation of Germline Alterations in Patients With Exocrine Pancreatic Neoplasms. Journal of the National Cancer Institute, 110(10), 1067–1074. https://doi.org/10.1093/jnci/djy024
Complete responses to PARP inhibition in germline or somatic *BRCA1/2*-mutated PDAC	Reiss, K. A., Mick, R., O'Hara, M. H., Teitelbaum, U., Karasic, T. B., Schneider, C., Cowden, S., Southwell, T., Romeo, J., Izgur, N., Hannan, Z. M., Tondon, R., Nathanson, K., Vonderheide, R. H., Wattenberg, M. M., Beatty, G., & Domchek, S. M. (2021). Phase II Study of Maintenance Rucaparib in Patients With Platinum-Sensitive Advanced Pancreatic Cancer and a Pathogenic Germline or Somatic Variant in BRCA1, BRCA2, or PALB2. Journal of clinical oncology : official journal of the American Society of Clinical Oncology, 39(22), 2497–2505. https://doi.org/10.1200/JCO.21.00003<br>Lowery, M. A., Kelsen, D. P., Stadler, Z. K., Yu, K. H., Janjigian, Y. Y., Ludwig, E., D'Adamo, D. R., Salo-Mullen, E., Robson, M. E., Allen, P. J., Kurtz, R. C., & O'Reilly, E. M. (2011). An emerging entity: pancreatic adenocarcinoma associated with a known BRCA mutation: clinical descriptors, treatment implications, and future directions. The oncologist, 16(10), 1397–1402. https://doi.org/10.1634/theoncologist.2011-0185<br>Shroff, R. T., Hendifar, A., McWilliams, R. R., Geva, R., Epelbaum, R., Rolfe, L., Goble, S., Lin, K. K., Biankin, A. V., Giordano, H., Vonderheide, R. H., & Domchek, S. M. (2018). Rucaparib Monotherapy in Patients With Pancreatic Cancer and a Known Deleterious BRCA Mutation. JCO precision oncology, 2018, PO.17.00316. https://doi.org/10.1200/PO.17.00316
Response to platinum-based chemotherapy in HRD PDAC (* indicates complete response(s) identified)	Pishvaian, M. J., Blais, E. M., Brody, J. R., Rahib, L., Lyons, E., De Arbeloa, P., Hendifar, A., Mikhail, S., Chung, V., Sohal, D., Leslie, S., Mason, K., Tibbets, L., Madhavan, S., Matrisian, L. M., & Petricoin, E., 3rd (2019). Outcomes in Patients With Pancreatic Adenocarcinoma With Genetic Mutations in DNA Damage Response Pathways: Results From the Know Your Tumor Program. JCO precision oncology, 3, 1–10. https://doi.org/10.1200/PO.19.00115<br>Wattenberg, M. M., Asch, D., Yu, S., O'Dwyer, P. J., Domchek, S. M., Nathanson, K. L., Rosen, M. A., Beatty, G. L., Siegelman, E. S., & Reiss, K. A. (2020). Platinum response characteristics of patients with pancreatic ductal adenocarcinoma and a germline BRCA1, BRCA2 or PALB2 mutation. British journal of cancer, 122(3), 333–339. https://doi.org/10.1038/s41416-019-0582-7<br>O'Reilly, E. M., Lee, J. W., Zalupski, M., Capanu, M., Park, J., Golan, T., Tahover, E., Lowery, M. A., Chou, J. F., Sahai, V., Brenner, R., Kindler, H. L., Yu, K. H., Zervoudakis, A., Vemuri, S., Stadler, Z. K., Do, R., Dhani, N., Chen, A. P., & Kelsen, D. P. (2020). Randomized, Multicenter, Phase II Trial of Gemcitabine and Cisplatin With or Without Veliparib in Patients With Pancreas Adenocarcinoma and a Germline BRCA/PALB2 Mutation. Journal of clinical oncology : official journal of the American Society of Clinical Oncology, 38(13), 1378–1388. https://doi.org/10.1200/JCO.19.02931<br>* Sonnenblick, A., Kadouri, L., Appelbaum, L., Peretz, T., Sagi, M., Goldberg, Y., & Hubert, A. (2011). Complete remission, in BRCA2 mutation carrier with metastatic pancreatic adenocarcinoma, treated with cisplatin based therapy. Cancer biology & therapy, 12(3), 165–168. https://doi.org/10.4161/cbt.12.3.16292<br>* Shimmura, H., Kuramochi, H., Jibiki, N., Katagiri, S., Nishino, T., & Araida, T. (2019). Dramatic response of FOLFIRINOX regimen in a collision pancreatic adenocarcinoma patient with a germline BRCA2 mutation: a case report. Japanese journal of clinical oncology, 49(11), 1049–1054. https://doi.org/10.1093/jjco/hyz141<br>* Park, J. H., Jo, J. H., Jang, S. I., Chung, M. J., Park, J. Y., Bang, S., Park, S. W., Song, S. Y., Lee, H. S., & Cho, J. H. (2022). BRCA 1/2 Germline Mutation Predicts the Treatment Response of FOLFIRINOX with Pancreatic Ductal Adenocarcinoma in Korean Patients. Cancers, 14(1), 236. https://doi.org/10.3390/cancers14010236
Rates of brain metastasis in patients with *BRCA*-mutated breast, ovarian, and pancreatic cancers	Song, Y., Barry, W. T., Seah, D. S., Tung, N. M., Garber, J. E., & Lin, N. U. (2020). Patterns of recurrence and metastasis in BRCA1/BRCA2-associated breast cancers. Cancer, 126(2), 271–280. https://doi.org/10.1002/cncr.32540<br>Garber, H. R., Raghavendra, A. S., Lehner, M., Qiao, W., Gutierrez-Barrera, A. M., Tripathy, D., Arun, B., & Ibrahim, N. K. (2022). Incidence and impact of brain metastasis in patients with hereditary BRCA1 or BRCA2 mutated invasive breast cancer. NPJ breast cancer, 8(1), 46. https://doi.org/10.1038/s41523-022-00407-z<br>Ratner, E., Bala, M., Louie-Gao, M., Aydin, E., Hazard, S., & Brastianos, P. K. (2019). Increased risk of brain metastases in ovarian cancer patients with BRCA mutations. Gynecologic oncology, 153(3), 568–573. https://doi.org/10.1016/j.ygyno.2019.03.004<br>Balendran, S., Liebmann-Reindl, S., Berghoff, A. S., Reischer, T., Popitsch, N., Geier, C. B., Kenner, L., Birner, P., Streubel, B., & Preusser, M. (2017). Next-Generation Sequencing-based genomic profiling of brain metastases of primary ovarian cancer identifies high number of BRCA-mutations. Journal of neuro-oncology, 133(3), 469–476. https://doi.org/10.1007/s11060-017-2459-z<br>Ratner, E., Bala, M., Louie-Gao, M., Aydin, E., Hazard, S., & Brastianos, P. K. (2019). Increased risk of brain metastases in ovarian cancer patients with BRCA mutations. Gynecologic oncology, 153(3), 568–573. https://doi.org/10.1016/j.ygyno.2019.03.004<br>Jordan, E. J., Lowery, M. A., Basturk, O., Allen, P. J., Yu, K. H., Tabar, V., Beal, K., Reidy, D. L., Yamada, Y., Janjigian, Y., Abou-Alfa, G. K., & O'Reilly, E. M. (2018). Brain Metastases in Pancreatic Ductal Adenocarcinoma: Assessment of Molecular Genotype-Phenotype Features-An Entity With an Increasing Incidence?. Clinical colorectal cancer, 17(2), e315–e321. https://doi.org/10.1016/j.clcc.2018.01.009
Response to PARP inhibition among patients with ovarian or breast cancer and CNS metastasis	Alizzi, Z., Roxburgh, P., Cartwright, D., McLaren, A., Park, S., Jones, R., Greening, S., Hudson, E., Green, C., Gray, S., Khalique, S., Karteris, E., & Hall, M. (2023). Description of a Retrospective Cohort of Epithelial Ovarian Cancer Patients with Brain Metastases: Evaluation of the Role of PARP Inhibitors in this Setting. Journal of clinical medicine, 12(7), 2497. https://doi.org/10.3390/jcm12072497<br>Bangham, M., Goldstein, R., Walton, H., & Ledermann, J. A. (2016). Olaparib treatment for BRCA-mutant ovarian cancer with leptomeningeal disease. Gynecologic oncology reports, 18, 22–24. https://doi.org/10.1016/j.gore.2016.10.004<br>Zhang, Z., Xu, M., Sakandar, A., Du, X., He, H., He, W., Li, D., & Wen, Q. (2022). Successful Treatment of a Patient With Brain Metastasis From Ovarian Cancer With BRCA Wild Type Using Niraparib: A Case Report and Review of the Literature. Frontiers in oncology, 12, 873198. https://doi.org/10.3389/fonc.2022.873198<br>Lam T. C. (2021). Comprehensive Genomic Profiling-Guided Niraparib Treatment of Triple-Negative Breast Cancer in a Patient With Extensive Brain Metastasis: Case Report and Literature Review. Journal of immunotherapy and precision oncology, 4(1), 16–20. https://doi.org/10.36401/JIPO-20-24<br>Pascual, T., Gonzalez-Farre, B., Teixidó, C., Oleaga, L., Oses, G., Ganau, S., Chic, N., Riu, G., Adamo, B., Galván, P., Vidal, M., Soy, D., Urbano, Á., Muñoz, M., & Prat, A. (2019). Significant Clinical Activity of Olaparib in a Somatic BRCA1-Mutated Triple-Negative Breast Cancer With Brain Metastasis. JCO precision oncology, 3, 1–6. https://doi.org/10.1200/PO.19.00012
