# Supplementary material for: A Somatic BRCA2-Mutated Pancreatic Adenocarcinoma With Sustained Exceptional Response to Modified FOLFIRINOX
Source: Oncologist. 2024 Feb 23;29(4):350–5. doi: 10.1093/oncolo/oyad315 (PMC10994267; doi:10.1093/oncolo/oyad315)
Supplement: oyad315_suppl_Supplementary_Tables_S2 [file oyad315_suppl_supplementary_tables_s2.pdf]

| Treatment | Clinical Trials Identifier | Phase | Status | Patient Population | Eligibility and Exclusion |
|---|---|---|---|---|---|
| Rucaparib and FOLFIRI | NCT03337087 | I/II | Active, Recruiting | -PDAC with HRD | -Metastatic disease<br>-Has not received prior systemic therapy for metastatic disease |
| Niraparib | NCT03601923 | II | Active, Recruiting | -PDAC with deleterious germline or somatic BRCA1/2, PALB2, CHEK2, or ATM mutations | -One line of prior therapy |
| Olaparib | NCT04348045 | II | Active, Recruiting | -PDAC with BRCAness profile | -Metastatic disease<br>-Stability or tumor response after 4 months of FOLFIRINOX (8 cycles) for metastatic disease |
| Niraparib and dostarlimab | NCT04493060 | II | Active, Recruiting | -PDAC with deleterious germline or somatic BRCA1/2, PALB2, BARD1, RAD51c, or RAD51d mutations | -Metastatic disease<br>-1-2 prior lines of systemic therapy for metastatic disease |
| Olaparib and pembrolizumab | NCT04666740 | II | Active, Recruiting | -PDAC with HRD or previous exceptional response to platinum-based therapy | -Metastatic disease<br>-Stable or responding disease on current first-line or second-line platinum treatment for metastatic disease |
| Olaparib | NCT04858334 | II | Active, Recruiting | -PDAC with germline or somatic BRCA1/2, PALB2 mutations | -Previously resected with no evidence of recurrent disease<br>-12 weeks of systemic, multi-agent chemotherapy |
